# Liposomes: Clinical Applications and Potential for Image-Guided Drug Delivery

**DOI:** 10.3390/molecules23020288

**Published:** 2018-01-30

**Authors:** Narottam Lamichhane, Thirupandiyur S. Udayakumar, Warren D. D’Souza, Charles B. Simone, Srinivasa R. Raghavan, Jerimy Polf, Javed Mahmood

**Affiliations:** 1 Department of Radiation Oncology, University of Maryland School of Medicine, Baltimore, MD 21201, USA; wdsouza@som.umm.edu (W.D.D.); CharlesSimone@umm.edu (C.B.S.I.); jpolf@umm.edu (J.P.); jmahmood@som.umaryland.edu (J.M.); 2 Department of Radiation Oncology, University of Miami Miller School of Medicine, Miami, FL 33136, USA; tudayakumar@med.miami.edu; 3 Department of Chemical and Biomolecular Engineering, University of Maryland, College Park, MD 20742, USA; sraghava@umd.edu

**Keywords:** liposomes, clinical applications, image guidance, radioisotopes, PET, SPECT, MRI

## Abstract

Liposomes have been extensively studied and are used in the treatment of several diseases. Liposomes improve the therapeutic efficacy by enhancing drug absorption while avoiding or minimizing rapid degradation and side effects, prolonging the biological half-life and reducing toxicity. The unique feature of liposomes is that they are biocompatible and biodegradable lipids, and are inert and non-immunogenic. Liposomes can compartmentalize and solubilize both hydrophilic and hydrophobic materials. All these properties of liposomes and their flexibility for surface modification to add targeting moieties make liposomes more attractive candidates for use as drug delivery vehicles. There are many novel liposomal formulations that are in various stages of development, to enhance therapeutic effectiveness of new and established drugs that are in preclinical and clinical trials. Recent developments in multimodality imaging to better diagnose disease and monitor treatments embarked on using liposomes as diagnostic tool. Conjugating liposomes with different labeling probes enables precise localization of these liposomal formulations using various modalities such as PET, SPECT, and MRI. In this review, we will briefly review the clinical applications of liposomal formulation and their potential imaging properties.

## 1. Introduction

Over the recent years of research innovation, drug delivery techniques have made a significant contribution to our understanding of drug tissue interactions. While many chemotherapeutic drugs and gene therapies have been developed in the last couple of decades, their efficacy is marred by toxicity and the inability to effectively reach the target site. The failure to deliver therapeutic agents at desired concentration to tumors derives from constraints that are innate to the tumor microenvironment or the bioactivity and bioavailability of therapeutic agents. Many tumors, such as pancreatic ductal adenocarcinoma (PDAC), harbor dense desmoplastic stroma that prevents therapeutic agents from effectively reaching the tumor cells [1]. In addition, increased interstitial fluid pressure (IFP) keeps the therapeutic agents at bay [2]. Low bioavailability due to elimination from circulation or biotransformation also contribute to impaired delivery of the therapeutic agents to the target site [3]. Mononuclear phagocyte system is very efficient at eliminating the therapeutic agents from circulation, hence impeding the delivery to the target site [2].

**Figure 1:**
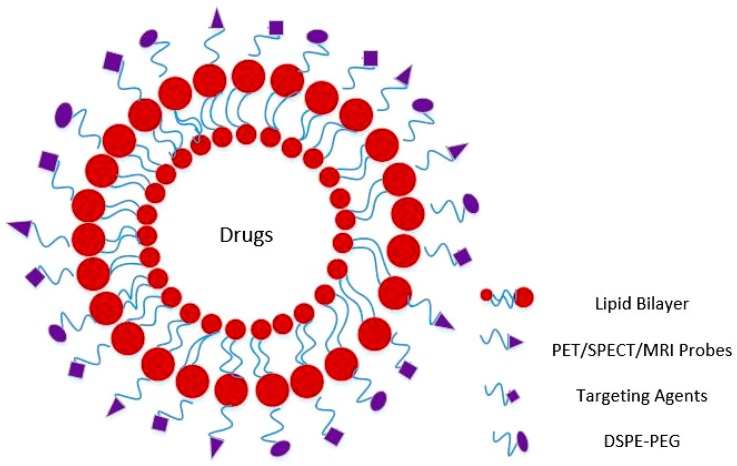
Schematics of a functionalized liposome.

Challenges in drug delivery due to the limited diffusion of drugs as a result of high interstitial pressure and, rapid clearance of intravenously administered drugs by the systemic circulation hamper the adequate uptake of drugs in tumor regions [4,5]. These challenges associated with drug delivery have spurred research in the field of “drug delivery” aimed at developing methods that aid in delivery of the drugs/molecules to the target sites for improved clinical outcomes. Circumventing such problems is a major challenge in drug delivery vehicles. Research has primarily focused on increasing bioavailability while at the same time improving the targeting of the therapeutic agents to the tumor site. Drug delivery methods such as liposomes, micelles, dendrimers, etc. [6,7,8,9] have been widely investigated for their potential in a wide range of clinical applications. However, liposomes are one of the potentially most promising drug delivery vehicles. Liposomes are lipid vesicles consisting of one or more concentric lipid bilayers enclosing an aqueous space (Figure 1). Liposomes present an attractive delivery system because of the flexibility of changing their chemical composition, structure and colloidal size by modifying the preparation methods [10]. Liposomes can therefore be manufactured with different size, ranging from several nanometers to micrometers. Flexibility of formulation of liposomes with varying the choice of bilayer components allows liposomes to be either rigid and impermeable or permeable and less stable [11]. Surface modifications allows liposomes to be tailored for both diagnostic, therapeutic, as well as image-guided drug delivery. These unique advantages of liposomes over other nanocarriers offer solutions to many limitations in diagnosis, delivery and treatment management of human diseases [12].

Despite their attractive characteristics, liposomal delivery systems also have some drawbacks. Liposomal systems can trigger an acute hypersensitivity syndrome known as complement activation-related pseudoallergy (CARPA) as a result of the innate immune response [13]. Conventional liposomes are cleared rapidly from the circulation by the macrophages that are located mainly in the liver, spleen and bone marrow [14,15,16]. The use of modified flexible hydrophilic polymers such as polyethylene glycol (PEG), which provide a protective hydrophilic layer on the surface of the liposome reduces the clearance of liposomes from the reticuloendothelial system (RES) [17,18,19]. Such pegylated liposomes, also known as “stealth liposomes”, have prolonged circulation time [20,21] and an improved pharmacokinetic profile compared with that of a free drug [22]. Owing to their many properties liposomes have been investigated pre-clinically and clinically on many fronts as a diagnostic and therapeutic tool. Molecular imaging using multimodal probes offers great potential for early and accurate diagnosis, real time monitoring of in vivo pharmacokinetics as well as detailed information of pathologies. Versatility in liposomal surface functionalization to attach different molecular probes enables multimodal imaging that can be exploited to derive accurate and precise assessment of hallmarks of various diseases. The purpose of this review is to focus mainly on the clinical application of liposomes and their potential use as imaging agents as well as in image guided drug delivery systems.

## 2. Clinical Applications of Liposomes

Clinically, liposomal formulations are used as carriers for biologically active molecules. Liposomes have been extensively studied in areas such as gene therapy [23] and drug delivery [24] due to their observed stability and favorable toxicity profile over traditional treatments. Liposomes can encapsulate biomolecules or drugs that are hydrophilic and increase their internalization and solubility through the lipid bilayers of the cells [25]. Research interest in liposomal formulations have increased significantly in the last decade and have been shown to be safer than viral vectors due to their low immunogenicity, more limited toxicity and their ability to carry larger cargo to the target sites [26,27]. Liposomal formulations have shown to accumulate in the target tissue—with enhanced bio-distribution [28]. Drug formulations with liposomes are approved for intravenous, intramuscular [29] and oral delivery [30] and the delivery is determined by the mechanism of drug loading, composition of the membrane, and the tumor microenvironment [31]. Clinically approved liposomal drugs—are pegylated variants to improve their time in circulation and protect the integrity of the drug from various degrading mechanisms active inside a tissue or cell.

Liposome-mediated drug delivery systems have been successfully translated into clinical settings [32]. These delivery systems are used in diverse medical fields, including anti-cancer, anti-fungal and anti-inflammatory drugs as well as therapeutic gene delivery. Many clinical products, e.g., Doxil^TM^, AmBisome^®^ and DepoDur [32] have been formulated using liposomes for clinical applications. Therapeutic use of various agents through alterations in their pharmacokinetics and pharmacodynamics were enhanced by encapsulation of drugs in liposomes. A number of liposome-based drug formulations are approved for human use and many additional products are currently being assessed in different clinical trials.

In 1995, Doxil^TM^ was first introduced in U.S., to treat ovarian cancer and AIDS-related Kaposi’s sarcoma [33]. Doxil^TM^, liposomal doxorubicin, was developed exploiting active loading by pH gradient method [34,35]. Further, DaunoXome^®^ was developed by NeXstar Pharmaceuticals (Boulder, CO, USA) for the delivery of daunorubicin, and was FDA approved in 1996 for the management of advanced HIV-associated Kaposi’s sarcoma. Other products since available include Mepact^®^ by Takeda Pharmaceutical (Deerfield, IL, USA), DepoCyt^®^ by SkyPharma Inc. (Belgravia, London, UK), Marqibo^®^ by Talon Therapeutics (San Francisco, CA, USA) and a fluorouracil, leucovorin combination with liposomes (Merrimack Pharmaceuticals Inc., Cambridge, MA, USA) therapy-based product for metastatic adenocarcinoma of the pancreas and Myocet^®^ by Elan Pharmaceuticals (San Francisco, CA, USA). Apart from cancer treatments, liposomal products were also developed for other diseases such as fungal infections (Amphotec^®^ and AmBisome^®^). Liposomes have become an important carrier systems for vaccine development leading to the development of vaccines such as Epaxal^®^ and Inflexal V^®^ for hepatitis and influenza, respectively.

### 2.1. Liposomal Formulations in Clinical Trials

Currently, there are a number of liposomal products undergoing clinical trials. In the past decade, extensive research on lipid carriers and liposomal formulations lead to the development of new liposome mediated drug delivery. Here, we highlighted some of the liposomal products in clinical trials (Table 1).

#### 2.1.1. Phase I

Liposomal formulations in phase I clinical trials include Grb-2, LEM-ETU, INX 0125 and 0076. We briefly discuss these trials under this section. Grb-2 is used for the treatment of breast cancer and leukemia.

Grb-2 liposomes (BP-100-1.01) inhibit the production of the growth factor receptor-bound protein-2. Grb-2 is a DOPC-integrated antisense oligonucleotide [61]. Grb-2 inhibits tumor cell proliferation due to its anti-neoplastic activities and the presence of antisense oligo-deoxy-nucleotide [62]. In a Phase 1 trial, Grb-2 is being studied for the treatment of relapsed or refractory acute myeloid leukemia and its safety, maximum tolerated dose, optimal therapeutic dose and anticancer activity is also being explored.

LEM-ETU liposomes is used in the management of various treatments including, leukaemia, breast, stomach, liver and ovarian cancers and are composed of DOPC, cholesterol and cardiolipin [63,64]. Compared to other liposomes, the presence of cardiolipin helps in drug entrapment and higher drug loading capability. Further, “NeoPharm’s NeoLipid liposome technology” developed these liposomes for the treatment of various cancers.

INX-0125 and INX-0076 are developed by Inex pharmaceuticals (Burnaby, British Columbia, Canada) and are undergoing a phase I clinical trial. Both of these liposomal formulations are composed of cholesterol and sphingomyelin (SM) and are developed for the treatment of advanced solid tumors [21]. Sphingosomal formulations enhance tumor targeting and also increase the duration of exposure for loaded anticancer agents without any increase in toxicity in preclinical trials [31]. INX-0125 has also been used in the treatment of Hodgkin’s and non-Hodgkin’s lymphoma. Based on phase I clinical trials, INX-0076 protects the drug from in vivo degradation and has been shown to accumulate at target sites thus increasing the efficacy of the drug [32].

#### 2.1.2. Phase II

Aroplatin™ (L-NDDP) is a chemotherapeutic platinum analogue (*cis*-bisneodecanoato-*trans*-*R*,*R*-1,2-diaminocyclohexane platinum II) and the first liposomal platinum formulation to enter into clinical trials; the loaded analogue is structurally similar to Eloxatin (Oxaliplatin; Sanofi Aventis, Bridgewater, NJ, USA). These platinum analogues have cytotoxic properties where, they form inter- and intra-strand cross-links of DNA, thereby inhibiting DNA synthesis in tumor cells [21,37]. Aroplatin™ analogues have been studied in preclinical models and found to be effective in inhibiting the emergence of liver metastasis of reticulosarcoma. This formulation reduced toxicity and, improved activity and bioavailability [65]. An early phase Phase I/II trial of Aroplatin™ was completed in 2002 for advanced solid malignancies, however, a phase I dose escalation trial was carried out in 2005 where a maximum tolerated dose was reached, followed by termination of this trial in spite of the clinical results obtained due to the inaccurate chemical composition along with the instability of the drug in liposomes [66].

LEP Easy-to-Use (LEP-ETU) is a novel delivery system of paclitaxel developed by NeoPharm, Inc. (Blainville, QC, Canada). Paclitaxel (taxol) is an anti-micro-tubular network agent that has been studied in different malignancies. Preclinical studies of liposome-entrapped paclitaxel (LEP) have demonstrated that LEP was associated with reduced toxicity while maintaining efficacy [40]. To enhance the solubility with Paclitaxel, this drug is formulated with polyoxyethylated castor oil that led to infusion-related hypersensitivity reactions. NeoPharm improved the safety profile by formulating LEP-ETU with a mixture of synthetic phospholipids and cholesterol that is necessary for administering higher doses than would commonly or safely be used with taxol. The results from phase I clinical trials of NeoPharm, showed LEP-ETU is better tolerated than taxol, as indicated by a higher maximum-tolerated dose (MTD).

OSI-211 liposomes (OSI Pharmaceuticals, Melville, NY, USA), a liposomal formulation of lurtotecan (LRT), are composed of a HSPC and cholesterol (2:1). OSI-211 is a topoisomerase I inhibitor, and is currently in Phase II clinical trials [67]. In pre-clinical studies the product demonstrated increased drug accumulation in tumor. Compared to topotecan, OSI-211 showed a similar toxicity profile in a randomized phase II clinical trial in relapsed ovarian cancer treatment [68]. Next-generation formulations OSI-211 and NX 211 were used in the treatment of ovarian and head & neck cancer.

Cisplatin is a highly effective chemotherapeutic agent against epithelial cancers [69]. To reduce the systemic toxicity of this agent, a liposomal pegylated formulation, Liposomal cisplatin (SPI-077), was developed. Phase I studies of SPI-77 began in 1995 at doses ranging from 40 to 420 mg/m^2^ [70]. Results from a Phase1 study demonstrated the ability to deliver ten-fold the amount of cisplatin per dose without encountering any toxicities beyond grade 1. Side-effects included mild gastrointestinal toxicity (nausea and vomiting), and mild anemia, muscle weakness (at doses of ≥320 mg/m^2^) and a case of infusion-related reaction [71].

#### 2.1.3. Phase III

Cyclophosphamide (CYP) is an alkylating agent broadly used as an anticancer chemotherapeutic agent for numerous malignancies [72,73,74]. The active carbonium ion produced by CYP reacts with nucleic acids and proteins of tumor cells. Pre-clinical studies have demonstrated that CYP entrapped in liposomes reduces non-specific toxicities and enhances anticancer effects [75,76]. Pre-clinical studies showed that encapsulation of CYP increased its mutagenicity resulting in accumulation of drug inside the cell and eventually causing chromosomal damage [77]. In a phase III trial of liposomal doxorubicin (Myocet) and a stereoisomer of doxorubicin (epirubicin) combined with cyclophosphamide as first-line therapy for metastatic breast cancer, it was shown that Myocet has modest but significant increase in efficacy than epirubicin [56].

Stimuvax^®^ (L-BLP-25 or tecemotide, Merck, Kenilworth, NJ, USA), is a therapeutic vaccine for cancers expressing Tumor-Specific Antigens (TSA) that integrates an antigenic lipo-peptide, i.e., tecemotide, in a liposomal delivery system. Mucin 1 (MUC1) is overexpressed in different cancer cells (breast, prostate, non-small cell lung cancer (NSCLC) and colorectal cancer) that are the target for tacetomide. Stimuvax, triggers a cellular immune reaction after targeting TSA, leading to immune rejection of tumors that have the MUC1 antigen [78]. Stimuvax^®^ looked promising in a randomized Phase II trial that led to a subsequent Phase III trial where Stimuvax^®^ was administered after chemotherapy and radiation therapy for locally advanced (stage III) NSCLC. In Phase II trials, Stimuvax^®^ showed a significant increase in life expectancy from 13.3 to 30.6 months and showed a positive outcome in stage III and IV NSCLC patients. However, in Phase III trials, Stimuvax^®^ did not meet its primary or secondary end-points, which led to the termination of the trial [46,79].

Liposomal formulation of cisplatin (CPT), Lipoplatin, is a FDA approved cytotoxic agent. Its mechanisms include DNA cross linking and inhibition of DNA synthesis. For pancreatic cancer the product used was Lipoplatin and for lung cancer was Nanoplatin [41]. Lipoplatin is composed of lipids including 1,2-dipalmitoyl-*sn*-glycero-3-phosphorylglycerol (DPPG), soy PC, monomethoxy-polyethylene-glycol-1,2-distearoyl-*sn*-glycero-3-phosphoethanolamine (MPEG-DSPE) lipid conjugate and cholesterol. The advantages of CPT incorporation in liposomes include increased cell permeability, longer half-life of the drug in circulation, and higher concentration of drugs in tumors [80]. Toxicities including renal and neuropathy were considerably reduced with lipoplatin compared to CPT given alone [81].

### 2.2. Liposomes and Image Guided Delivery

Imaging plays an integral part in modern precision and individualized medicine. Wide applications of imaging such as monitoring drug delivery, accurate diagnosis of diseases, determining response to therapy, and guiding minimally invasive procedures are some of the applications of imaging in clinic. However, traditional imaging modalities such as computed tomography (CT), positron emission tomography (PET), magnetic resonance imaging (MRI), and single photon emission computed tomography (SPECT) all suffer from target specificity, limiting their clinical utility. Nanoparticles with their versatility in surface functionalization provide opportunities to enhance target specificity and label nanoparticles with various isotopes that enables them to act as contrast agents.

Radionuclide imaging offers unique strengths in cancer diagnosis. Use of radiolabel liposomes could act as a companion diagnostic. Pre-administration of radiolabeled liposomes and quantifying the uptake of liposomes in target sites can help in determining drug responders vs. non-responders. This will also help in determining an optimal dose to predict a potential therapeutic response. Hence, insufficient accumulation may represent lack of therapeutic response. In such scenarios, the liposomes can be altered to better manage patients [82]. The majority of radionuclide imaging is carried out using PET and SPECT. Multimodality imaging such as MRI and CT provides detail anatomical information, however they are not capable of providing disease states. On the other hand, PET and SPECT can measure chemical changes pre- and post-treatment intervention [83]. The potential of functionalization of liposomes offers an advantage of engineering liposomes to target cancer cells for the use in radionuclide imaging of malignant lesions. For this purpose, the radionuclide can be directly conjugated on the surface of the liposomes or can be encapsulated in the aqueous core (Figure 1). Radiolabeling of liposomes can be achieved using various labeling procedures. Passive encapsulation of radionuclide, labeling the liposomal membrane or labeling the preformed liposomes by loading the radionuclide using an ionophore or chelator are different labeling ways. Application of radiolabeled liposomes for clinical settings require high incorporation efficiency and good retention of radiolabel agents. Remote afterloading of liposomal formulations had shown most efficient labeling and best radiolabel retention [82]. Various PET and SPECT radioisotopes have been utilized to conjugate with liposomes to evaluate their clinical utility in multimodal imaging. Quantitation of liposomes generated by SPECT or PET images also enables non-invasive data analysis over time [84,85].

### 2.3. PET Imaging

Quantitative imaging with nanoparticle provides accurate and precise assessment of pathologies in cancer care and opens up the pathway for personalized precision medicine. PET imaging is an outstanding tool to derive a quantitative measure and real time monitoring of the biomarker uptake in vivo. Various researchers have studied the feasibility of PET imaging by conjugating PET isotopes in long circulating liposomes [83,86,87,88,89,90,91]. Wong et al. [92] evaluated the clinical utility of ^64^Cu (T_1/2_ = 12.7 h) labeled liposomes as diagnostic and therapeutic tools to measure tumor volume, as a contrast agent, and compared the biodistribution of ^64^Cu-labeled liposomes to the clinical tracer ^18^F-FDG. In this study, Wong et al. reported that the estimates of tumor diameter were comparable between ^64^Cu-liposomes and ^18^F-FDG and heterogeneity of uptake with both tracers. The feasibility of image contrast with both tracers was also reported. Another study by Seo et al. [93] studied two ^64^Cu-radiolabeled lipids of different acyl chains (1 mol % DSPE vs. 1 mol % DPPE) in long circulating liposomes and presented the differences of stability of these lipids using PET imaging. Remote loading method of ^64^Cu into liposomes using 2-hydroxyquinoline to transport ^64^Cu through the membrane to the copper chelator was studied by Petersen et al. [89]. Seo et al. [84] also looked into the pharmacokinetics of ^89^Zr (T_1/2_ = 78.4 h) labeled liposomes over the extended period of time in a murine model. Zirconium (Zr) with a relatively longer decay has been exploited as a potential isotope for PET imaging to study the pharmacokinetics for an extended period. In this study, Seo et al. reported that the location of ^89^Zr label altered the clearance rate of intracellularly trapped radioactivity and suggested optimization of chelator and attachment strategies for future studies to use ^89^Zr for image based pharmacokinetics. Perez-Medina et al. [94] also reported on ^89^Zr labeling of liposomes using two different approaches for pharmacokinetic and biodistribution. They studied two different approaches of ^89^Zr labeling using click labeling and surface chelation and observed that surface chelation is superior in terms of stability and in vivo performance. Marik et al. [95] synthesized ^18^F (T_1/2_ = 110 min) radiolabeled diglyceride, 3-[^18^F]-fluoro-1,2-dipalmitoylglycerol [^18^F] fluorodipalmitin ([^18^F] FDP) and its conjugation to synthesize ^18^F labeled liposomes. Marik et al. [95] reported the feasibility of PET imaging using both the radiolabeled [^18^F] FDP and liposome-incorporated [^18^F] FDP. Freely injected [^18^F] FDP had the highest uptake in the liver, spleen and lungs. Liposomal [^18^F] FDP remained in blood circulation at near-constant levels for at least 90 min, with a peak concentration near 2.5% ID/cc. Hansen et al. studied the EPR effect in 11 canine cancer patients with spontaneous solid tumors using ^64^Cu-loaded liposomes by PET/CT imaging. In this study, Hansen et al. included different tumor types and showed tumor dependent differences in accumulation and heterogeneity of uptake of the radiotracers. The study showed that the squamous cell carcinomas had the highest EPR effect as compared to other type of tumors [96]. Luo et al. studied the effects of phorphyrin-phospholipid inclusion in ^64^Cu-radiolabeled doxorubicin containing stealth liposomes (Dox-PoP). Luo et al. showed that Dox-PoP liposomes accumulated passively into tumor using both PET ad fluorescence imaging. Luo et al. also demonstrated strong primary tumor growth inhibition with a single treatment of chemophototherapy using 665 nm light (200 J/cm^2^) [97].

Recent work on PET labeling has also focused on the optimization procedures using longer lived isotope such as ^52^Mn (T_1/2_ = 5.6 days). A study by Jensen et al. [98] evaluated the optimal protocols on ^52^Mn labeling through both remote-loading and surface labeling. In this study, Jensen et al. reported that the labeling efficiencies of ^52^Mn with both labeling procedures were similar; however, the plasma half-life of surface conjugated ^52^Mn liposomes were shorter than remote loading. The popularity of PET in routine clinical practice due to its ability to diagnose diseases, more accurately stage malignancies, evaluate treatment response, detect CT-occult metastatic lesions and various other characteristics have established this imaging modality as a workhorse of nuclear medicine procedures. Tremendous efforts on developing image drug delivery systems are still ongoing with the intent of obtaining better diagnosis and therapy of different diseases. PET labeling of liposomes is an effort at circumventing some of these challenges which requires precise formulation, surface architecture and functionalization, and accurate design of drug loading procedures.

### 2.4. SPECT Imaging

SPECT is another commonly used imaging modality in nuclear medicine procedures. Like PET, SPECT imaging also requires an injection of molecular probes that are labeled with radionuclides. ^111^In (T_1/2_ = 2.81 days), ^99m^Tc (T_1/2_ = 6 h) and ^67^Ga (T_1/2_ = 78.26 h) represent some of the SEPCT isotopes that are used to label liposome to enable SPECT signal. Espinola et al. [99] studied the organ distribution of ^99m^Tc and ^111^In oxine labeled multilamellar lipid vesicles in 1978. In that study, Espinola et al. reported that constant distribution of ^111^In-oxine labeled vesicles was observed throughout 72 h as compared to ^99m^Tc labeled vesicles that showed continuous leakage of radioactivity from the involved organs. Ogihara et al. [100,101,102,103] studied the differential uptake of ^67^Ga and ^67^Ga labeled liposomes in tumors and inflammatory regions in rats. In that study, Ogihara et al. reported that positively charged liposomes preferentially delivered ^67^Ga to the tumor compared with granulation tissue, suggesting that ^67^Ga labeled liposomes are able to discriminate tumor and inflammatory lesions. Proffitt et al. [104] successfully imaged EMT6 tumors in BALB/c mice using In-111 nitrilotriacetic acid loaded liposomes. Biodistribution studies performed using three different liposomes with different charges showed the highest uptake with neutral ^111^In nitrilotriacetic acid loaded liposomes. Turner et al. [105] developed ^111^In-labeled liposomes and studied dosimetric effects of these liposomes in 24 patients. Tumor was observed in 22 out of 24 patients in the scans. However, Turner et al. reported homogenous uptake of these liposomes in the liver and spleen with scans obtained 24 and 48 h post injection which is typical of nanoparticles. In clinical studies, Kubo et al. reported tumor imaging of seven patients using ^111^In-labeled V-liposomes. Results from that study revealed increased activity in the tumors of four patients [106]. Furthermore, rapid blood clearance of liposomes with homogenous uptake in the liver and spleen was observed. One of the challenges in nuclear medicine is the localization of infectious and inflammatory lesions. In order to evaluate the potential of liposome to visualize foci of infection and inflammation, Boerman et al. [107] investigated ^111^In-labeled pegylated liposomes in *S. aureus* infection induced in rats using gamma imaging. As a control, ^111^In-IgG images were used as a comparison to ^111^In-liposomes. Abscesses were visualized within an hour of ^111^In-liposome post injection. Contrast between the infectious focus and the background increased with time. The ratio of abscess to background was significantly higher (*p* < 0.04) with ^111^In-liposomes as compared to ^111^In-IgG [108]. ^99m^Tc labeled liposomes were also investigated from the same group to evaluate their feasibility to detect infection and inflammation. The authors reported that ^99m^Tc liposomes preferentially accumulated in abscesses [107]. Another clinical study by Harrington et al. investigated the effective targeting of solid tumors in 17 patients with locally advanced cancers using ^111^In-labeled PEGylated liposomes with tumor seen in 15 of these patients. In 12 of the 17 patients, the tumor was clearly visible in the whole body scan; in an additional 3 patients that included 2 gliomas and 1 cervical cancer, SPECT scans of the regions of interest were required to identify the tumors [109]. As a representation of the study, Harrington et al. presented gamma camera images of three patients with three different cancers at 72 h post ^111^In-DTPA labeled pegylated liposomes that showed clear depiction of tumors in all three images [109]. Molecular imaging using SPECT enabling liposomes are increasingly being investigated to diagnose various diseases including inflammation, plaques, cancers and many more [110,111,112,113,114,115]. Molecular imaging using radiolabeled nanoparticles is also aimed at facilitating better delivery, in vivo pharmacokinetics monitoring, as well controlled release. In lieu of this, there has been limited research into radiolabeling liposomes with dual isotopes as a hybrid model [116,117]. A recent study by Lamichhane et al. also looked into ^111^In-labeled liposomes as a drug delivery vehicle for a ^18^F-labeled carboplatin derivative. This hybrid liposome construct was used to acquire dual modality imaging of a liposome vehicle using SPECT and drug using PET. This allowed for determining the pharmacokinetics and in vivo behavior of drug and vehicle distinctively using two different imaging modalities [118].

### 2.5. MRI Imaging

MRI is the most powerful non-invasive imaging modality that offers high soft tissue contrast, spatial resolution, and penetration depth [119]. Despite the relatively high soft tissues contrast with MRI images, in some cases it does not allow for enough image contrast to diagnose the pathology of interest. Such cases require the use of contrast agents to improve the contrast-to-noise ratio by shortening the spin-lattice T_1_ and/or spin-spin T_2_ relaxation times of the water protons within the region of interest [120,121,122]. There is an increased interest in using liposomes as contrast agents for MRI due to their tunable properties and lower toxicities as compared to the currently used contrast agents such as gadolinium. Liposomes have long been proposed as a vehicle to deliver paramagnetic ions by entrapping them inside them to reduce the systemic toxicity and potential to increase the contrast to noise ratio [123]. Multimodality imaging offers an additional advantage of retrieving information based on different modalities. Researches have also assessed bimodal detection of tumor using optical/MRI tagged liposomes. Ding et al. studied the effectiveness of targeting by evaluating folate receptor targeted fluorescent paramagnetic liposomes for tumor imaging. In their study, folate targeting was verified by measuring the uptake of folate conjugated liposomes using confocal microscopy and comparing the uptake to the untargeted liposomes. The MR image of HeLa cells incubated with folate conjugated Gd containing liposomes was also brighter than that of HeLa cells incubated with only Gd-DTPA [124]. Local delivery of drug to the tumor region increases the therapeutic ratio of chemotherapeutic agents. Releasing the payload encapsulated inside liposomes and quantifying the amount of release provides feedback on optimizing liposomes for specific purposes. Encapsulating chemotherapeutic agents with gadolinium and releasing these agent in tumor using high intensity focused ultrasound offers promise in local delivery and MRI guided therapy [112,125,126]. A similar approach of encapsulating non-Gd-containing T_1_-MR contrast agents based on Fe-succinyl deferoxamine (Fe-SDFO) has been studied as a safe alternative [127]. In this study, Kneepkens et al. reported that the amount of doxorubicin delivered to the tumor correlated with the rate of relaxation change. Another challenge in drug delivery is the blood brain barrier. Convection enhanced delivery (CED) of drugs enables higher concentration of drugs insitu as compared to systemic administration. Nordling-David et al. studied the delivery of temozolomide (TMZ) encapsulated liposomes in conjunction with GD-DTPA liposomes using CED to treat glioblastoma multiforme (GBM). This study reported that the co-infusion of pegylated Gd-DTPA liposomes and TMZ-liposomes by CED in GBM bearing rats, resulted in enhanced tumor detection with longer residence time than free Gd-DTPA. Treatment of GBM-bearing rats with either TMZ solution or TMZ-liposomes resulted in greater tumor inhibition and significantly higher survival; with no significant difference in survival with liposome formulation as compared to TMZ alone [128]. Shao et al. studied the difference of MR contrast using prophyrin-phospholipd and amino functionalized porphyrin phospholipid in BALB/c mouse. In this study Shao et al. synthesized manganese conjugated liposomes using 2-[1-hexyloxyethyl]-2-devinyl pyropheophor-bide-a (HPPH), a porphyrin derivative. This derivative was amine modified to derive N-HPPH-lipid which was also used to synthesize manganese conjugated liposomes. With the amino modification, Shao et al. observed 150% higher T_1_ relaxivity (mms)^−1^, increase to 2.46 for Mn-N-HPPH liposomes from 0.98 form Mn-HPPH liposomes [129].

## 3. Conclusions

Liposomes have been explored for various diseases ranging from cancer treatment to pain management. Advantages of using liposomal formulations include: (1) the properties of these liposomes like pharmacokinetics and pharmacodynamics are easily maneuverable, (2) improved bioavailability and (3) reduced toxicity. Different liposomal formulations are made for various applications such as temperature sensitive liposomes, cationic liposomes and liposomal vaccines. Collectively, these liposomal formulations have the ability to enhance or to overcome the limitations of conventional therapies. Furthermore, liposomes have shown great promise in their design to label them with molecular probes for imaging. Exploitation of liposomal characteristics to improve the target specificity and encapsulation can achieve significant therapeutic efficacy. Many liposomal formulations have successfully translated to clinical applications after extensive research on their efficacy and preclinical trials have demonstrated a greater impact on patients with various ailments, thereby improving the quality of life. Designing such liposomes with imaging probes can further enable real time delivery, monitoring and assessment of biological signatures that can ultimately lead to effective and personalized treatment.

## Figures and Tables

**Table 1:** Liposomes under different phases of clinical trials.

	Phase I		Phase II		Phase III		References
	Drug/Name	Agent/Target	Drug/Name	Agent/Target	Drug/Name	Agent/Target	
**1**	BP1001	Antisense protein/Grb-2	Aroplatin	L-NDDP/Platinum	Arikace	Amikacin/Ribosomal inhibitor	[36,37,38]
**2**	INX-0125	Sphingomyelin/cholesterol	LEP-ETU	Paclitaxel/microtubule	Lipoplatin	Cisplatin	[39,40,41]
**3**	INX-0076	Topotecan Sphingosomes	OSI-211	Lurtotecan/antineoplastic	Liprostin	PGE-I/Prostaglandin Receptor	[32,42,43]
**4**	LiPlaCis	Cisplatin/solid tumors	S-ANNA	Annamycin/TOPO II	Stimuvax	Tecemotide/Immunosuppressant	[44,45,46]
**5**	LEM-ETU	Mitoxantrone/TOPO II inhibitor	S-CKD602	TOPO I inhibitor	TAN5	T4 endonuclease V	[26,47,48]
**6**	SGT-53	p-53	SPI-077	Cisplatin/	Thermodox	Doxorubicin/antimitotic	[49,50,51]
**7**	LDF01	Cationic liposomes/Microvessels	Tretnoin	Retinoids/Skin disease	MiR-122	MicroRNA-122/HCV	[52,53,54]
**8**	Atu027	siRNA/Solid tumors	Irinotecan SN-37	Camptothecin/DNA damage	Cyclophosphamide	Nitrogen mustard/antineoplastic	[55,56,57]
**9**	Navelbine	Vinca alkaloid/Immunosuppressant	Taxol	Paclitaxel/microtubule	CPX-351	Cytarabine:daunorubicin/DNA polymerase inhibitor	[58,59,60]
